# Advances in CpG Island Methylator Phenotype Colorectal Cancer Therapies

**DOI:** 10.3389/fonc.2021.629390

**Published:** 2021-02-26

**Authors:** Xiaofei Zhang, Wenjun Zhang, Pingan Cao

**Affiliations:** ^1^Department of Medical Oncology, Dalian University Affiliated Xinhua Hospital, Dalian, China; ^2^Department of Colorectal Surgery, Dalian University Affiliated Xinhua Hospital, Dalian, China

**Keywords:** colorectal cancer, CpG island methylator phenotype, chemotherapy, targeted therapy, DNA methylation inhibitor

## Abstract

With the aging of the population, the incidence of colorectal cancer in China is increasing. One of the epigenetic alterations: CpG island methylator phenotype (CIMP) plays an important role in the incidence of colorectal cancer. Recent studies have shown that CIMP is closely related to some specific clinicopathological phenotypes and multiple molecular phenotypes in colorectal cancer. In this paper, the newest progress of CIMP colorectal cancer in chemotherapeutic drugs, targeted agents and small molecular methylation inhibitors are going to be introduced. We hope to provide potential clinical treatment strategies for personalized and precise treatment of colorectal cancer patients.

## Background

Colorectal cancer (CRC) is one of the most common malignant gastrointestinal tumors that seriously threaten the human health. It has a high incidence and mortality worldwide, and the incidence of CRC in China is also increasing year by year (1). Tumorigenesis of colorectal cancer is multistep and complex process involving genetic and epigenetic alterations. Epigenetic alterations refer to changes in gene expression without changing in the DNA sequence, leading to silencing of transcriptional genes or inactivating DNA repair genes and tumor suppressor genes (2). Over past 30 years, more and more studies indicate that epigenetic changes including DNA methylation, histone modification, nucleosome localization and small non-coding RNAs etc. play a key role in the tumorigenesis of colorectal cancer (3).

In recent years, DNA methylation modification has been extensively studied. In humans, DNA methylation to form 5-methylcytosine occurs mainly at the cytosine residues’ fifth carbon position of CpG dinucleotides. Around 60–80% of CpG cytosines are methylated in human somatic cells, however CpG islands are regions with a high frequency of CpG sites mostly located near the transcription start site of promoter genes which are constitutively unmethylated. In 1999, Toyota et al. (4) first proposed a novel subset of CRCs positive for CpG island methylator phenotype (CIMP) that extensively displayed multiple cancer specific genes promoter DNA hypermethylation at some specific set of CpG islands in CRC tissues. CIMP is now considered as a distinct molecular subtype of sporadic CRC which is the initial event of the serrated neoplasia pathway in CRC’s tumorigenesis (5). CpG island methylator phenotype (CIMP) is mediated by DNA methyltransferases (DNMTs) which promotes hypermethylation in promoter associated CpG-rich regions of tumor suppressor genes which are inactivated by transcription, leading to development and progression of CRC (6). Although there is no consensus on the definition of CIMP and no methodology has been proven to be superior to another (7–9), CIMP has been still recognized as a hotspot research direction of colorectal cancer in these past 20 years.

## Clinicopathologic and Molecular Characteristics in CIMP CRC

CIMP in CRC was confirmed not only tightly associated with some specific clinicopathologic phenotypes, but also closely related to many molecular characteristics. In 2006, Weisenberger and colleagues (10) recommended a novel and sensitive panel of CIMP including five specific genes’ promoter DNA hypermethylation markers to identify CIMP-positive CRCs. The study demonstrated that CIMP-positive was significantly related to female gender, proximal location, MSI-H status, *MLH1* methylation, *BRAF* mutation, and *KRAS* mutation. In their further large population-based sample analysis, older age, family history of CRC and NSAIDs using before diagnosis related to CIMP-positive were additionally observed. Furthermore, smoke and overweight statistically associated with only female CIMP-positive CRCs were reported (11). Ogino and colleagues (12) selected different promoter loci as CIMP panel markers to identify CIMP-High, CIMP-Low and non-CIMP phenotypes. Follow-up experiments showed that CIMP-Low tumors different from CIMP-High and non-CIMP tumors were tightly correlated to male gender and *KRAS* mutation (13). In further CIMP subgroup analysis, CIMP-positive tumors independent of MSI status were significantly associated with mucinous or signet ring cell morphology, tumor infiltrating lymphocytes (TIL), peritumoral lymphocytes (PLS), presence of Crohn-like infiltrates, tumor necrosis, tumor cell sheeting, and poor differentiation (14). Interestingly, low level intake of folate was proven to be associated with a trend towards an increased risk of non-CIMP-High colon tumors (15). Recent years, CIMP status was also reported to be positively correlated with F. nucleatum, the gut microbiome component in CRC (16, 17). Using more advanced methods or meta-analysis to evaluate the relationship between CIMP status and the clinicopathological and molecular characteristics in CRC, the conclusions obtained by Guinney et al. (18) and Advani et al. (19) were similar to those of Weisenberger and Ogino. In the past 20 years, CIMP-positive tumors were accepted as a consensus which were positively correlated with female, proximal location, MSI-H status, *BRAF* mutation and mucinous histology. These epidemiology associations help us explore the underlying cancer prevention and treatment strategies.

## CIMP and Colorectal Cancer Treatment

A growing number of studies suggest that CIMP might be a potential epigenetic predictor or prognostic biomarker contributed to individualized and precise treatment of colorectal cancer patients (20, 21).

### CIMP and Chemotherapy

As the most widely used chemotherapeutic drug for colorectal cancer, 5-fluorouracil (5-FU) principally acts as a thymidylate synthase (TS) inhibitor by interrupting DNA replication. In 2007, Shen et al. (22) investigated CIMP status in 188 advanced CRCs who received 5-FU based chemotherapy and found that the median survival in the CIMP-positive subset was 6 months versus 17 months in CIMP-negative subset (P < 0.001) and two-year survival rate was 8% in the CIMP-positive group versus 28% in the CIMP-negative group (**Table 1**). In multivariate analysis, CIMP-positive cases had a significantly shorter survival (hazard ratio, HR=2.9; P < 0.0001). Jover et al. (23) studied 196 stage II-III CRCs and found that CIMP-positive CRCs did not benefit from 5-FU based adjuvant treatment. The disease-free survival (DFS; log-rank=0.02) of CIMP-positive patients receiving adjuvant 5-FU based chemotherapy was lower than that of CIMP-negative patients. In CIMP-negative CRCs, adjuvant 5-FU based chemotherapy significantly prolonged DFS (log-rank=0.00001). However, it failed to improve DFS (log-rank=0.7) in CIMP-positive CRCs. Multivariate analysis showed adjuvant 5-FU based treatment was not an independent predictor of prognosis in CIMP-positive CRCs (HR=0.8; 95% confidence interval, CI=0.3–2.0). Min and colleagues (24) reached the opposite conclusion by an independent Asian population clinical trials. They performed 124 stage II–III CRCs and reported that CIMP-high CRCs (n=17; 3-year recurrence-free survival, RFS: 100%) who received 5-FU based regimen after surgery had significantly better RFS than those accepted surgery alone (n=7; 3-year RFS: 71.4%; P=0.022). Furthermore, Rijnsoever et al. (25) considered that CIMP-positive status was an independent significant predictor for the survival benefit treated with adjuvant 5-FU based chemotherapy in CRCs. They evaluated CIMP-positive status in 103 stage III CRCs treated with surgery alone and 103 cases treated with surgery plus adjuvant 5-FU based chemotherapy and provided evidence that CIMP positive CRCs could receive longer cumulative survival time from surgery plus 5-FU based treatment than surgery alone (P=0.002). However, CIMP-negative CRCs displayed no these association (P=0.6). For investigating the response of CIMP subtypes to 5-FU based chemotherapy, in the light of MSI, *BRAF* and *KRAS* molecular classification, Murcia et al. (26) classified 324 stage II-III CRCs into six study subgroups and reported that microsatellite stability (MSS), *BRAF* wild-type and CIMP-negative subtype CRCs had a longer time of DFS when they treated with 5-FU based chemotherapy (log rank P = 0.003 for *KRAS* mutation subgroup and P < 0.001 for *KRAS* wild-type subgroup). In a multivariate analysis, they found only MSS, *BRAF* wild-type, *KRAS* wild-type and CIMP-negative subgroup CRCs independently displayed significant benefit with 5-FU based chemotherapy (HR = 2.06, 95% CI 1.24–3.44, P = 0.005). In these independent clinical trials for stage II-III CRCs, the efficacy of 5-FU based regimen on CIMP or CIMP subgroup patients still seemed controversial. In my opinion, the main reason for the inconsistent results may be related to the different methylation markers and technologies used to define CIMP status, which may lead bias to distinguish the real CIMP-positive patients. For example, MethyLight based on real-time PCR with specific probe was considered much more reliable than MSP to define CIMP status. In Min and Murcia’s studies, the same method is used to define CIMP status. Furthermore, we should not merely consider the efficacy of 5-FU alone for adjuvant chemotherapy. the influence of oxaliplatin, irinotecan and radiotherapy should also be well evaluated. The application of 5-FU plus oxaliplatin- and irinotecan-based chemotherapy increased nearly double survival benefit of CRCs. FOLFIRI (5-FU, calcium folinate and irinotecan) and FOLFOX (5-FU, calcium folinate and oxaliplatin) were two important cytotoxic combined regimens treated with CRCs. Researchers confirmed that first-line FOLFIRI regimen and second-line FOLFOX regimen performed the same clinical efficacy comparing with reverse sequential regimen for metastatic CRCs (35). Zhang et al. (27) investigated 125 metastatic CRCs treated with combination chemotherapy and demonstrated that CIMP-positive metastatic CRCs showed significantly benefit from irinotecan-based regimen followed by FOLFOX rather than the reverse sequential regimen. Median progression free survival (mPFS) was prolonged 8.6 months (mPFS=15.2 vs 6.6 months, P = 0.043) and median overall survival (mOS) was extended 8 months (mOS=20.8 vs 12.8 months, P = 0.11). Shiovitz and colleagues (28) assessed the association between survival of 615 stage III colon cancer patients receiving 5-FU and leucovorin alone or with irinotecan (IFL) after surgery and CIMP status. The results showed that CIMP-positive patients receiving IFL versus FU/LV treatment were tended to increase OS (69 vs. 56%; 95% Cl: 0.37–1.05; P = 0.07), especially for the mismatch repair-intact (MMR-I) subgroup (P = 0.01), while CIMP-negative patients did not have this trend (HR = 1.38; 95% Cl: 1.00–1.89; P = 0.049). CIMP-positive patients with stage III CRC could obviously benefit from irinotecan-based regimen. This might be largely driven by MMR-I tumors which was associated with improved OS (28). For patients with metastatic colorectal cancer, irinotecan-based regimen also improved the survival of CIMP-positive patients. We speculated that stable DNA damage response (DDR) related genes (e.g. RECQ helicases) could enhance the hypersensitivity of CIMP-positive CRC to irinotecan. Of course, this need to be confirmed by further basic study. Previous studies have shown that demethylation treatment can activate multiple cancer cell signaling pathways not only allow the use of less toxic doses of irinotecan but also improve the efficacy of it (36, 37). It was seemed that CIMP-positive status was a potential biomarker to predict irinotecan-based chemotherapy regimen for CRCs.

**Table 1:** CIMP status and chemotherapy in colorectal cancer.

First Author and Year	Overall Population	Positive (High) Number(Rate)	Markers	Definition of CIMP Status	Methods	TNM Stage	Evaluated Treatment	Prognosis (Evaluation index)
Shen L. (22)	185	28(15%)	CIMP(*MINT1, MINT31, p14^ARF^, p16^INK4a^*)	≥2/4 loci	COBRA MSP	IV	5-FU	CIMP-positive Worse Survival (Survival)
Jover R. (23)	302(196)	93	CIMP (*CACNA1G, NEUROG, RUNX3, SOCS1, MLH1*)	≥3/5 loci	Bisulfite Pyrosequence	II-III	5-FU	CIMP-positive No Benefit (DFS)
Min BH. (24)	245	124	CIMP(*CACNA1G , IGF2, NEUROG, RUNX3, SOCS1*)	≥3/5 loci	MethyLight assay	II-III	5-FU	CIMP-positive Benefit (3-year RFS)
Van Rijnsoever M. (25)	206	103	CIMP (*CDKN2A, MINT-2, MDR1*)	≥2/3 loci	MSP	III	5-FU	CIMP-positive Benefit (Cumulative Survival)
Murcia O. (26)	878(324)	210	CIMP(*CACNAG1, SOCS1,RUNX3, NEUROG1, MLH1*)	≥3/5 loci	MethyLight assay	II-III	5-FU	CIMP-negative with MSS, BRAF wild-type and KRAS wild-type Benefit (DFS)
Zhang XF., 2016 (27)	125	27 (21.6%)	CIMP(*CACNA1G , IGF2, NEUROG, RUNX3, SOCS1*)	≥3/5 loci	MSP	IV	FOLFOX, irinotecan-based regimen	Irinotecan-based regimen followed by FOLFOX Benefit (PFS, OS,RR)
Shiovitz S. (28)	615	316/299	CIMP(*CACNA1G , IGF2, NEUROG, RUNX3, SOCS1*)	≥3/5 loci	MethyLight assay	III	5-FU and leucovorin, IFL	IFL regimen especially for MMR-I subgroup Benefit (OS, DFS)
Cohen SA. (29)	292	292	CIMP(*CACNA1G , IGF2, NEUROG, RUNX3, SOCS1*)	≥3/5 loci	MethyLight assay	II-III	mFOLFOX6, XELOX	No significant difference (DFS, OS)
Han SW. (30)	322	322	CIMP (*CACNA1G, CDKN2A, CRABP1, IGF2, MLH1, NEUROG1, RUNX3, SOCS1*)	CIMP1 (CIMP-High) ≥5/8 loci CIMP2 (CIMP-Low) 1-4 loci	MethyLight assay	II-III	FOLFOX	No significant difference (3-year DFS)
Gallois C. (31)	1867	275(14.7%)	CIMP(*CACNA1G , IGF2, NEUROG, RUNX3, SOCS1*)	≥3/5 loci	MSP	III	FOLFOX, FOLFOX plus Cetuximab	Poor efficacy from FOLFOX regimen (OS, DFS, SAR)
Cha Y. (32)	153	7 (4.5%)	CIMP (*CACNA1G, CDKN2A, CRABP1, IGF2, MLH1, NEUROG1, RUNX3, SOCS1*)	CIMP-High ≥5/8 loci CIMP-Low 1-4 loci	MethyLight assay	IV	FOLFOX, XELOX, SOX, FOLFIRI, XELIRI, Capecitabine alone	Poor efficacy from chemotherapy (PFS, OS,RR)
Bae JM. (33)	1370	951 (531/365/49/6)	CIMP (*CACNA1G, CDKN2A, CRABP1, IGF2, MLH1, NEUROG1, RUNX3, SOCS1*)	CIMP-P2 (CIMP-High) ≥7/8 loci CIMP-P1 (CIMP-Low) 5-6 loci	MethyLight assay	I-IV	5-FU and leucovorin alone, FOLFOX, FOLFIRI, Radiotherapy alone	CIMP-P2 Benefit from chemotherapy (CSS, RFS)
Overman MJ. (34)	21	15	CIMP (*MLH1, p16, p14, MINT1, MINT2, MINT31*)	≥2/6 loci	MSP	IV	Nab-paclitaxel	No Benefit (PFS, OS)

CIMP, CpG island methylator phenotype; COBRA, combined bisulfite restriction analysis; MSP, methylation-specific polymerase chain reaction;

DFS, disease-free survival; RFS, relapse-free survival; SAR, survival after recurrence; OS, overall survival; RR, response rate; PFS, progression-free survival;

CSS,cancer-specific survival; FOLFOX, 5-FU, leucovorin and oxaliplatin; FOLFIRI, 5-FU, leucovorin and irinotecan; IFL, 5-FU, leucovorin and irinotecan;

XELOX, oxaliplatin and capecitabine; SOX, oxaliplatin and S-1; XELIRI, Irinotecan and capecitabine.

Cohen et al. (29) studied CIMP status from 292 stage II-III CRCs who received adjuvant modified FOLFOX6 (mFOLFOX6) or XELOX (capecitabine and oxaliplatin). There was no significant difference in OS between CIMP-positive and CIMP-negative patients (HR=1.27; 95%CI: 0.58–2.80; P=0.55). Han et al. (30) analyzed CIMP status based on 322 stage II–III CRCs who received adjuvant FOLFOX chemotherapy and found that CIMP-high status CRCs had no significantly associated with 3-year DFS comparing with CIMP-Low or CIMP-negative CRCs (P=0.31). Although study illustrated that CIMP status could not be a significant prognostic biomarker for adjuvant oxaliplatin-based chemotherapy regimens in stage II-III CRCs, there seemed to be a tendency that the efficacy of oxaliplatin in CIMP-positive patients was worse than that in CIMP-negative patients. In a large-population clinical cohort research, Gallois et al. (31) investigated CIMP status of 1867 stage III CRCs who treated with adjuvant FOLFOX or FOLFOX plus cetuximab regimen and found that the OS (HR = 1.46; 95% CI: 1.02–1.94; P = 0.04) and survival after recurrence (SAR; HR = 1.76; 95% CI: 1.20–2.94; P < 0.0004) of CIMP-positive patients significantly shortened, but no significant difference of DFS (HR = 1.15; 95% CI: 0.86–1.54; P = 0.34) were observed. Cha et al. (32) divided 153 metastatic CRCs treated with systemic chemotherapy into three CIMP groups. The results were demonstrated that the OS were significantly different among the three CIMP groups with a median of 9.77, 22.2, and 35.7 months for the high, low and negative groups, respectively (P< 0.001). In 5-FU and oxaliplatin first-line chemotherapy (n=128), the median OS was 6.77, 23.8, and 37.9 months for the high, low and negative groups, respectively (P<0.001), while the median PFS was 1.83, 7.87 and 9.97 months, respectively (P=0.002). CIMP-high cases were significantly associated with worst efficacy of therapy. In 5-FU and irinotecan second-line chemotherapy (n=86), only the median OS was shown a significant difference according to the CIMP status with values of 2.90, 13.4, and 20.4 months for the high, low and negative groups, respectively (P<0.001). The CIMP-high status was considered as a negative prognostic factor for metastatic CRCs received with chemotherapy. Bae and colleagues (33) inspected 1,370 stage I–IV CRCs treated with surgery and/or chemotherapy. Compared with CIMP-P1 (CIMP-L), CIMP-negative CRCs showed better 5-year cancer-specific survival (CSS; HR=0.47; 95% CI: 0.28–0.78) and better 5-year RFS (HR=0.50; 95% CI: 0.29–0.88). The CIMP-H CRCs displayed best 5-year CSS from chemotherapy was observed, however no such trend was found in no chemotherapy analysis. Multiple clinical studies seemed to reach an agreement on CIMP-positive CRCs associated with poor survival, but failed to display a prognostic value of CIMP-positive CRCs who were treated with oxaliplatin-based adjuvant chemotherapy regimen. Albumin-bound paclitaxel is an anti-microtubule drug, which interferes with the rearrangement of microtubules, leading to the cessation of mitosis, thus inhibiting the growth of cancer cells. Overman et al. (34) reported a phase II clinical trial which enrolled 21 CIMP-high metastatic CRCs and no efficacy of nab-paclitaxel was observed. Oxaliplatin-based or paclitaxel-based chemotherapy might be indirectly affected by CRCs’ CIMP status.

The DNA repair gene O6-methylguanine-DNA methyltransferase (*MGMT*) promoter methylation is a frequent and early event in colorectal tumorigenesis which was considered benefit from alkylating agents such as temozolomide (TMZ) (38). In a phase II study, TMZ showed a modest activity and achieved an average 10% RR in heavily pretreated metastatic CRC patients with *MGMT* hypermethylation (39). A recent study was shown that irinotecan and TMZ (TEMIRI) combination regimen was reached the primary end point in irinotecan-sensitive, *MGMT* methylated and MSS pretreated metastatic CRC patients (40). Six out of 25 patients achieved PR (ORR=24%; 95% CI, 11–43%). The mPFS and mOS were 4.4 and 13.8 months, respectively. All patients with *MGMT*-positive IHC were non-responders. Consistently, patients with *MGMT*-negative/low tumors had a significantly longer mPFS than others (6.9 vs 2.0 months; HR = 0.29, 95% CI 0.02–0.41; P = 0.003). The reason of the efficacy of TEMIRI regimen for metastatic CRC patients with MGMT methylation and absent/low might be the inhibition of topoisomerase II enhances the cytotoxicity of alkylating agents.

### CIMP and Targeted Therapy

Cetuximab, an epidermal growth factor receptor (EGFR) inhibitor, is an IgG1 monoclonal antibody specifically targeting EGFR overexpression and widely used in metastatic CRCs. In the 2 studies described above, Zhang et al. (27) demonstrated that the PFS of CIMP-positive and *KRAS* wild-type CRCs who treated with cetuximab was shorter than that of CIMP-negative CRCs with *KRAS* wild-type (mPFS, 2.1 vs. 5.1 months, P = 0.11) and objective response rate (ORR) was also decreased (20.0 vs. 24.4%, P = 0.90). Although this study did not show statistical significance, it seemed to suggest that CIMP-positive phenotype might be a biological negative predictor of efficacy of anti-EGFR antibody (**Table 2**). Also Gallois and colleagues (31) concluded that the application of cetuximab in CIMP-positive stage III CRCs bought a non-significant trend of negative efficacy. Ouchi et al. (41) analyzed 97 *KRAS* wild-type metastatic CRCs received anti-EGFR antibody by advanced genome-wide DNA methylation technique and divided patients into highly methylated epigenotype (HME), intermediate methylated epigenotype (IME), and low methylated epigenotype (LME). The results were shown that ORR (35.7 vs 6.3%, P = 0.03), disease control rate (DCR; 75 vs 31.3%, P = 0.005), PFS (HR = 0.22; 95% CI, 0.13–0.57; P < 0.001) and OS (HR = 0.19; 95% Cl, 0.06–0.54; P < 0.001) were significantly better in LME subgroup CRCs compared with HME subgroup CRCs. Although merely one study displayed statistical significance, we still believe that CIMP-positive phenotype is a negative efficacy indicator for *KRAS* wild-type metastatic CRCs who received anti-EGFR antibody. First, CIMP was significantly associated with the right side CRC which was considered as an independent negative prognostic factor to cetuximab therapy (42, 43). A clear mechanistic understanding to explain the worse outcome of CIMP-positive patients is currently lacking. EGFR promoter hypermethylation status may be responsible and has been reported to be more relevant than primary right colon in the prediction of negative response to anti-EGFR therapy in patients with metastatic CRCs (44). Secondly, we speculated that some hypermethylation genes increased the resistance of anti-EGFR antibody and lead to drug resistance. Demethylation agents were given to CIMP-positive CRCs which might reverse the resistance and improve patients’ survival. This is a potential research direction to change the therapeutic strategy of metastatic CRC in the future.

**Table 2:** CIMP status and targeted therapy in colorectal cancer.

First Author and Year	Overall Population	Positive (High) Number (Rate)	Markers	Definition of CIMP Status	Methods	TNM Stage	Evaluated Treatment	Response (Evaluation index)
Zhang XF. (27)	125	27 (21.6%)	CIMP(*CACNA1G, IGF2, NEUROG, RUNX3, SOCS1*)	≥3/5 loci	MSP	IV	Cetuximab or Panitumumab	A tendency of negative efficacy (PFS, OS)
Gallois C. (31)	1867	275(14.7%)	CIMP(*CACNA1G, IGF2, NEUROG, RUNX3, SOCS1*)	≥3/5 loci	MSP	III	Cetuximab	A tendency of negative efficacy (OS, DFS, SAR)
Ouchi K. (41)	97(45/52)	17(37.8%) 17(32.7%)	485577 CpG sites	HMCC: CIMP-H LMCC: CIMP-L	Genome-wide DNA methylation	IV	Cetuximab or Panitumumab	Negative efficacy (OS, PFS, DCR,ORR)

CIMP, CpG island methylator phenotype; MSP, methylation-specific polymerase chain reaction; HMCC, Highly methylated colorectal cancer;

LMCC, Low methylated colorectal cancer; DFS, disease-free survival; PFS, progression-free survival; SAR, survival after recurrence; OS, overall survival;

DCR, disease control rate; ORR, objective response rate.

### CIMP and DNA Methylation Inhibitor

DNMT1, DNMT3A and DNMT3B are the canonical cytosine-5 DNMT enzymes. Their functions include not only the establishment and maintenance of DNA methylation patterns, but also the regulation of multiple gene functions, including transcriptional silencing, transcriptional activation and post-transcriptional regulation (45). Most widely studied DNA methylation inhibitors were 5-azacytidine (Azacitidine, 5-Aza-CR), 5-aza-2’-deoxycytidine (Decitabine, 5-Aza-CdR) and guadecitabine (SGI-110) formed irreversible complexes with DNMTs by substituting methylated cytosine targets during DNA replication, leading to the depletion of the enzyme and cytosine during cell division, passive DNA demethylation, tumor suppressor genes’ re-expression, proliferation control and carcinogenesis process inactivation (46, 47). It was suggested that CIMP-positive CRCs might potentially benefit from the treatment with DNA methylation inhibitors alone or combination (48, 49). Garrido-Laguna et al. (50) confirmed 20 patients with *KRAS* wild-type metastatic CRC receiving sequential decitabin and panitumumab were well tolerated in phase I/II clinical trials (**Table 3**). Two of patients previously received cetuximab had a partial response (PR). Ten patients had stable disease (SD). Although CIMP status was not inspected, the combination of DNA methylation inhibitor and panitumumab was shown activity in *KRAS* wild-type metastatic CRCs previously treated with cetuximab. Overman and colleagues (51) demonstrated that 26 metastatic CRCs treated with oxaliplatin-based regimen refractory receiving azacitidine and CAPOX (capecitibine and oxaliplatin) in phase I/II clinical trials. The results displayed 14 patients were CIMP-high. However, not correlated with SD and PFS. In this clinical study, CIMP status was failed in validating as a predictive factor for DNA methylation inhibitor. Azad et al. (52) enrolled 47 metastatic CRCs treated with azacitidine and histone deacetylase inhibitor entinostat in phase II clinical study. Although patients were tolerable with combination epigenetic therapy, no significant clinical activity was observed. Recently, Jansen and colleagues (53) investigated nine eligible patients with pretreated unresectable liver-predominant metastases from solid tumors and evaluated the safety and antitumor activity of administrating decitabine by hepatic arterial infusion (HAI) in phase I clinical trial. Four out of all patients suffered from CRC who were more heavily pretreated with chemotherapy. Results were shown decitabine could be safely administered by HAI. No objective response was observed, while after treatment, the upregulation of cancer testis antigens (CTAs) expression indicated decitabine combined with immunotherapy could be candidate treatment in the further study. From the above studies, it seemed that DNA methylation inhibitors combined with the traditional chemotherapy were shown no significant effective, while the efficacy of combination anti-EGFR antibody or immunotherapy seemed to be worth looking forward to. Although low-dose DNA methylation inhibitors show demethylation and promote apoptosis, inhibiting DNMT alone may not be sufficient to induce durable and robust transcriptional gene re-expression. DNA methylation inhibitors as immune modulators have been consequently considered inducing CTAs expression in CRC which stimulate cytotoxic T-cell responses and antitumor immunity (54). Furthermore, DNA hypermethylation of tumor-infiltrating immune cells or their ligands (e.g. PD-1, CTLA-4, TIM-3, TIGIT, PD-L1, and galectin-9) leading to tumor evasion from host immunosurveillance could be major contributors to the upregulation of immune checkpoints (55). Combining immunotherapy to evaluate the activity of DNA methylation inhibitors is the most promising research direction in the future. In addition, epigenetic therapies included not only DNA methylation inhibitors, but also HDAC inhibitor, BET inhibitor and EZH2 inhibitor. Different epigenetic alterations should be given appropriate interventions. Development of these therapies will provide exciting opportunities for novel and improved therapeutic interventions in CRC (56).

**Table 3:** Clinical trials involving DNA methylation inhibitors listed on.www.clinicaltrials.gov.

Trial ID	Status	Enrollment	Interventions	Conditions	Purpose
NCT01193517	Completed	26/14	5-Azacitidine and CAPOX	Metastatic CRC	Phase I: To find the highest tolerable dose of azacitidine combined with CAPOX that can be given to patients with metastatic CRC. Phase II: To study efficacy and safety of combination can help to control CIMP metastatic CRC.
NCT02260440	Completed	31	5-Azacitidine and Pembrolizumab	Chemo-refractory metastatic CRC	Phase II: To evaluate the anti-tumor activity, safety, and tolerability of Pembrolizumab in combination with azacitidine in subjects with chemo-refractory mCRC without any further standard treatment options.
NCT01105377	Completed	47	5-Azacitidine and Entinostat	Metastatic CRC	Phase II: To study how well giving azacitidine together with entinostat works in treating patients with metastatic CRC.
NCT02959437	Completed	70	5-Azacitidine with Pembrolizumab and Epacadostat	Advanced or metastatic solid tumors	Phase I/II: Dose-escalation assessment to evaluate the safety and tolerability of the combination therapies.
NCT03182894	Withdrawn	0	5-Azacitidine with Pembrolizumab and Epacadostat	chemo-refractory MSS mCRC	Phase IB/II: To evaluate the safety, tolerability and anti-tumor efficacy of epacadostat in combination with pembrolizumab plus azacitidine in patients with chemo-refractory MSS mCRC.
NCT02811497	Active	28	5-Azacitidine and Durvalumab	MSS CRC PR-OC ER+ and HER2- BC	Phase 2: To assess the antitumor activity of azacitidine in combination with durvalumab in advanced solid tumors.
NCT02316028	Completed	11	Decitabine	Unresectable Liver Metastatic CRC	PhaseI: Toxicity and efficacy of Decitabine delivered by HAI in patients with non-resectable liver metastatic CRC.
NCT00879385	Completed	21	Decitabine and Panitumumab	Advanced metastatic CRC	To evaluate the safety and feasibility of the sequential use of decitabine with panitumumab for KRAS wild type tumors in the second or third line treatment of advanced mCRC.
NCT01882660	Terminated	88	Decitabine	Stage II-III CRC	Phase I: To assess in patients with primary colon cancer whether short-course pre-operative treatment with decitabine can increase Wnt target gene expression as measured in resected tumors compared to pretreatment biopsies.
NCT01966289	Active	18	SGI-110 with CY and GVAX	Metastatic CRC	Phase I: Difference in CD45RO+ TILs measured by immunohistochemistry in pre and post-treatment tumor biopsies from mCRCs. To evaluate the efficacy, safety and tolerability of treatment.
NCT01896856	Completed	96	SGI-110 combined with irinotecan versus regorafenib or TAS-102	Previously treated mCRC	Phase I: To determin MTD of SGI-110 combined with irinotecan. Phase II: To evaluate the efficacy of SGI-110 and irinotecan versus the standard of care regorafenib or TAS-102.
NCT03576963	Recruiting	45	SGI-110 Plus Nivolumab	Refractory CIMP+ mCRC	Phase Ib: To study the side effects and best dose of SGI-110 Plus Nivolumab. Phase II: To assess the efficacy of SGI-110 Plus Nivolumab

CIMP, CpG island methylator phenotype; CAPOX, capecitabine and oxaliplatin; MSS, microsatellite stable; CRC, colorectal cancer;

ER, estrogen receptor; BC, breast cancer; PR-OC, platinum resistant epithelial ovarian cancer type II; NSCLC, non-small cell lung cancer;

HAI, hepatic arterial infusion; GVAX, allogeneic colon cancer cell vaccine; CY, cyclophosphamide; TILs, tumor infiltrating lymphocytes;

MTD, maximum tolerated dose.

## Conclusion

In summary, we reviewed the latest progress of CIMP CRCs’ characteristics and treatment. We clearly realize that CRCs with CIMP phenotype are tightly related to the pathological features of female, older age and right side colon, as well as molecular characteristics of *BRAF* mutation and MSI-H status. Certainly, with the wide application of next generation sequencing technology, more accurate method to distinguish CRCs’ CIMP status will be are constantly emerging. For chemotherapy, CIMP-positive CRCs were potentially benefit from irinotecan-based regimen rather than oxaliplatin-based regimen. For targeted therapy, negative efficacy from anti-EGFR antibodies seems to be associated with CIMP-positive CRCs. However, the mechanism of these phenomena needs to be further explored in the future. Clinicians are increasingly aware of the importance of CIMP phenotype in CRC. A various of DNA methylation inhibitors alone or especially combination with immunotherapy are undergoing clinical trials. These frontier studies provide potential individualized precise treatment strategies for patients with CRC.

## Data Availability Statement

All data collected, generated, or analyzed during this study are included in this published article.

## Author Contributions

The authors have contributed equally to this work. All authors contributed to the article and approved the submitted version.

## Funding

This work was financially supported by the Guidance Plan of Natural Science Foundation of Liaoning Province (2019-ZD-0302), Dalian Youth Science and Technology Star Plan (2019RQ096), and Clinical plus Excellence Project (2020ZYA003) from Shanghai Nucleic Acid Chemistry and Nanomedicine Key Laboratory.

## Conflict of Interest

The authors declare that the research was conducted in the absence of any commercial or financial relationships that could be construed as a potential conflict of interest.

## Abbreviations

5-Aza-CdR, 5-aza-2’-deoxycytidine; 5-Aza-CR, 5-azacytidine; CACNA1G, Calcium voltage-gated channel subunit alpha 1G; CI, Confidence interval; CIMP, CpG island methylation phenotype; CRABP1, Cellular retinoic acid binding protein1; CRC, Colorectal cancer; CSS, Cancer-specific survival; CTAs, Cancer testis antigens; DCR, Disease control rate; DFS, Disease-free survival; DNMT, DNA methyltransferase; EGFR, Epidermal growth factor receptor; FOLFIRI, 5-FU, calcium folinate and irinotecan; FOLFOX, 5-FU, calcium folinate and oxaliplatin; HAI, Hepatic arterial infusion; HME, Highly methylated epigenotype; HR, Hazard ratio; IEL, Intraepithelial lymphocytes; IFL, 5-FU and leucovorin alone or with irinotecan; IGF2, Insulin like growth factor 2; IME, Intermediate methylated epigenotype;

LME, Low methylated epigenotype; MDR1, Multi-drug resistance 1; mFOLFOX6, Modified FOLFOX6; MMR-I, Mismatch repair-intact; MSI, Microsatellite instability; MSP, Methylation-specific polymerase chain reaction; MSS, Microsatellite stability; MTHFR, Methylenetetrahydrofolate reductase; NEUROG, Neurogenin 1; ORR, Objective response rate; OS, Overall survival; PFS, Progression free survival; PLS, Peritumoral lymphocytes;

PR, Partial response; RFS, Recurrence-free survival; RUNX3, Runt-related transcription factor 3; SAM, S-adenosylmethionine; SAR, Survival after recurrence; SCNAs, Somatic copy number alterations; SD, Stable disease; SGI-110, Guadecitabine; SOCS1, Suppressor of cytokine signaling 1; TIL, Tumor infiltrating lymphocytes; TS, Thymidylate synthase; XELOX, Capecitabine and oxaliplatin.
